# The prevalence of HIV in the sudden, unexplained and unexpected death population at the Pretoria Medico-Legal Laboratory

**DOI:** 10.4102/sajhivmed.v17i1.424

**Published:** 2016-05-31

**Authors:** Neil K. Morris, Lorraine du Toit-Prinsloo, Lynne Webber, Gert Saayman

**Affiliations:** 1 Department of Forensic Medicine, University of Pretoria, South Africa; 2 Department Medical Virology, University of Pretoria, South Africa

## Abstract

**Purpose:**

To determine the prevalence of HIV in the sudden, unexplained and unexpected (SUU) death population admitted to the Pretoria Medico-Legal Laboratory.

**Methods:**

This study was conducted at the Pretoria Medico-Legal Laboratory. Blood samples were obtained from decedents who died suddenly and/or unexpectedly, during autopsy, by a forensic pathologist. Sample collection continued until 100 valid samples were analysed for HIV antibodies. The data collected included demographic details and case-related information.

**Results and Conclusion:**

SUU deaths accounted for 14% of all cases admitted to the Pretoria Medico-Legal Laboratory. The HIV prevalence in the SUU deaths was 43%, which is 17% higher than the general mortuary population in Pretoria (*p* = 0.0045). The majority of these deaths were due to respiratory disease processes, with 12 cases having HIV/TB co-infection.

## Introduction

There are numerous definitions for sudden deaths. All of the definitions include two main parameters. They relate to the time from the onset of symptoms and signs, and the time of death.^1^ These times vary between zero, 1, 6 and 24 h.^1^ The WHO defines a sudden death as a death occurring within 24 hours after the onset of symptoms.^2^ Byard classified these deaths into three categories: those individuals who were apparently completely well and died suddenly; those who were mildly unwell; and those with known serious disease processes but were stable on treatment.^1^ The admission of cases of sudden, unexpected and/or unexplained deaths (SUU-Deaths) to medico-legal mortuaries is a well-known worldwide phenomenon, and in many of these cases an inadequate history is present at the time of the investigation.^3^

In South Africa, the performance of medico-legal autopsies is regulated by the *Inquests Act* (No. 58 of 1959). The definition of unnatural deaths is in the Regulations Regarding the Rendering of Forensic Pathology Service (R636) in accordance with Chapter 11 of the *National Health Act* (No. 61 of 2003). These include cases ‘where the death is sudden and unexpected, or unexplained, or where the cause of death is not apparent’.

SUU-Deaths are considered to be some of the most challenging autopsies that can be conducted by pathologists.^3^ In many of these cases, it is difficult to ascertain whether or not the person died from a disease process found at autopsy or as a result thereof.^4^

HIV can cause death of an individual in numerous different ways including AIDS, accelerated cardiovascular disease, malignancies and opportunistic infections.^5^ AIDS is regarded as being the number one cause of death in HIV patients.^6^ Tseng et al. indicated that sudden cardiac deaths in HIV-positive individuals accounted for 86% of all cardiac deaths with a mean rate of 2.6 per 1000 person-years.^6^

Antiretroviral therapies have been associated, in a number of studies, with the increased risk of cardiovascular disease.^7,8^ It was documented that: ‘Increased exposure to protease inhibitors is associated with an increased risk of myocardial infarction, which is partly explained by dyslipidaemia’.^9^ TB and HIV co-infection may also be linked to SUU-Death. TB and HIV co-infected patients have a five times greater risk of dying within 2 years of receiving TB treatment.^10^ Other studies have determined that 31% of all new TB cases in the African region can be attributed to HIV infection: ‘TB was the cause of 11% of all adult AIDS-related deaths’. In South Africa, it was estimated that there were 2 million co-infected adults in 2003.^11,12^

South Africa has a jaded past with misinformation and the stigmatisation of HIV-positive individuals.^13,14^ Due to these social factors, the disclosure of an accurate medical history is not forthcoming in cases of SUU-Death. This ends up hindering the medico-legal investigation of the death.

South Africa had a population of 49 320 500 people as of mid-2009, and 10.6% of the population was HIV-positive.^15^ The burden of the disease on the state is large and costly. In order to minimise the consequences of the pandemic, every possible chance to understand and refine the information, used to make important prevention and treatment decisions, needs to be implemented and assessed.

This study seeks to provide relevant statistical information on the HIV prevalence rate in the SUU-Death population of the Pretoria Medico-Legal Laboratory (MLL). The results of this study could also provide additional insight into the epidemiology of the virus and the SUU-Death population. This can facilitate a protocol change in the handling of the SUU-Death population admitted to the Pretoria MLL. This would be to implement standardised HIV testing in SUU-Death cases, which, in turn, will have budgetary and policy implications for the Forensic Pathology Service (FPS).

## Materials and methods

### Study population

This study was conducted at the Pretoria MLL. Pretoria is the capital city of South Africa with a population of approximately 2 553 648.^16^ The Pretoria MLL admits approximately 2500 cases each year, and full autopsies are conducted in more than 90% of the cases admitted. A previous study conducted at the Pretoria MLL indicated that the HIV prevalence of the general mortuary population was 26%.^17^ The SUU-Death population makes up 14% of the general mortuary population. Samples were collected until the study had obtained 100 valid results.

### Method

Blood samples were obtained from decedents who died suddenly and/or unexpectedly. This was done during autopsy, by a forensic pathologist. Sample collection continued until 100 valid samples were analysed. A study and funding limitation was that only 100 samples were to be collected.

The processing of blood samples was done by the following standardised testing procedures developed and used in the NHLS Tshwane virology research department:

- After centrifugation of whole blood, the serum was used to run the test in order to minimise the effect that haemolysed blood has on test strips.
- The screening test used was the Determine™ HIV-1/2 Ag/Ab Combo assay, and the confirmatory test used was the HIV Combi, Cobas E, Elecsys and Modular (Roche).^18^

#### Exclusionary criteria

The following cases were excluded: if age was less than 9 months because of the possible residual positive reaction to the mothers’ antibodies in the child^19,20^; and if it was not possible to obtain a sample due to post-mortem changes (decomposition) or charring of the remains.

#### Data collection and analysis

The data collected included demographic details and case-related information. Confidentiality was ensured, and the study proposal was approved by the University of Pretoria’s Faculty of Health Sciences Research Ethics Committee and the MSc Committee.

The statistical analysis was done in conjunction with the Department of Statistics at the University of Pretoria. The null hypothesis was that the prevalence of HIV in the Pretoria MLL SUU-Death population should not deviate from that of the general Pretoria MLL population. The statistical programme SAS^®^ was used for the analysis of data.

## Results

The data collection took approximately 10 months to complete in 2009. A total of 130 samples were collected, of which 100 samples yielded a valid HIV result. The remaining 30 samples when tested provided an invalid result on the test (the absence of a test result). This was discussed in a previous study, and it could be caused by an increased post-mortem interval.^17^

### Demographic details

Table 1 lists the results of the HIV screening and confirmatory testing that was done at the NHLS Tshwane medical virology laboratory. A total of 30 invalid test results were documented; these translate into 25% of all samples. Of the viable samples, 43 (43%) were HIV-positive and 57 (57%) were HIV-negative.

**TABLE 1:** Total sudden, unexplained and unexpected death cases and HIV prevalence information in the Pretoria Medico-Legal Laboratory.

Result	Number of cases	%	Number of valid samples	%
HIV-positive	43	32	43	43
HIV-negative	57	43	57	57
Invalid	30	25	-	0
**Total**	**130**	**-**	**100**	**-**

Table 2 lists the gender and race distribution of the HIV-positive cases. Twenty-one per cent (21 cases) of the SUU-Death sample population was female and 79% (79 cases) was male. Of the female demographic fraction, the HIV-positive cases made up 62% (13 cases) of the total valid samples. In the male demographic fraction, the HIV prevalence rate was 38% (30 cases).

**TABLE 2:** Gender and race demographics for sudden, unexplained and unexpected death cases.

Demographic parameters	Variable	HIV-positive	%	HIV-negative	%	Total valid samples
Gender	Female	13	62	8	38	21
	Male	30	38	49	62	79
**Total**		**43**	**-**	**57**	**-**	**100**
Race	Asian	0	0	2	100	2
	Black	40	51	39	49	79
	Mixed race	0	0	0	0	0
	White	3	16	16	84	19
**Total**		**43**	**-**	**57**	**-**	**100**

The race demographics for the SUU-Death cases are listed in Table 2. Of the total sample population, Asian individuals made up 2% (2 cases), with none of them being documented as HIV-positive. Black population group made up 79% (79 cases), and the prevalence of HIV was 51% (40 cases). No individuals classified as ‘mixed race’ were documented in this study. White South Africans made up 19% (19 cases) of the SUU-Death population, and the HIV prevalence is 16% (3 cases) in this group.

**FIGURE 1:**
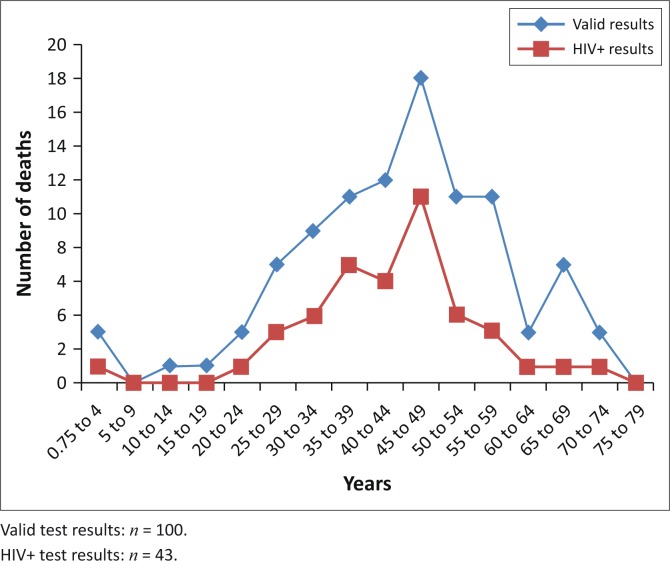
Age distribution of the valid sudden, unexplained and unexpected death samples and the HIV-positive results.

The age of individual cases in this study ranged from 9 months to 74 years (Figure 1). Data were divided into categories with a 5-year interval between the groupings. Positive HIV immune assay results were obtained only from samples in the 9 months – 74 years range, resulting in a 43% rate of infection across the entire Pretoria MLL SUU-Death population. The average age for the valid sample population was 43 years. The average age for females was 39.0 years and for males 44.9 years. The average age in the males from the black population group was 45.5 years and in the males from the white population group was 42.6 years. The average age in the females from the white and black population groups was 37.2 years and 39.3 years, respectively.

### Cause of death

The causes of death are summarised in Table 3 below. The majority of the deaths were due to respiratory disease processes (31 in total) with 12 cases of TB, 15 cases of bronchopneumonia, 2 pulmonary thrombo-embolism cases and 1 each of asthma and small-cell lung carcinoma (19 cases of pulmonary disease). Cardiovascular disease processes included six cases of ischaemic heart disease, two cases of myocarditis and nine cases of cardiomyopathy. In the central nervous system, one case each of meningitis and sudden unexpected death in epilepsy (SUDEP) and two cases of haemorrhagic cerebrovascular incident were present; only one case was signed out as being due to AIDS. In 11 cases which presented as sudden unexpected deaths, unnatural causes of death was identified, which included acute alcohol poisoning, carbon monoxide poisoning and trauma.

**TABLE 3:** Causes of death.

Cause of death	Number of cases
Cardiovascular diseases	17
Pulmonary diseases	19
Tuberculosis	12
Central nervous system	4
AIDS	1
Genito-urinary system	2
Gastro-intestinal tract	6
Natural causes	6
Non-natural causes	11
Pregnancy-associated disease	1
Unascertained	14
Under investigation	7

*n* = 100.

The prevalence of both the general populations HIV statistics (previously conducted study^17^) and the SUU-Death populations HIV statistics were compared in Table 4 with a chi-squared analysis, which yielded a statistically significant *p*-value of 0.0045.^17^

**TABLE 4:** Chi-square analysis of the general population HIV statistics and sudden, unexplained and unexpected death HIV prevalence statistics.

General mortuary population’s HIV prevalence %	General mortuary population’s HIV prevalence	SUU-Death population’s HIV prevalence %	Chi-square analysis (*p*)
26	17	43	0.0045

## Discussion

The HIV prevalence in the SUU-Death population at the Pretoria MLL is 43%. This is significantly higher than the HIV prevalence in the general mortuary population at the Pretoria MLL (26%), translating into a 17% difference in prevalence between the two groups.^17^ This was determined to be statistically significant. White South Africans account for 18% of the general mortuary population and their HIV prevalence was 8% in the general mortuary population.^17^ However, in the SUU-Death population they accounted for an unexpected 19% of the population and had an HIV prevalence of 16%. Black South Africans accounted for 76% of the general mortuary population with an HIV prevalence of 32%.^17^ In the SUU-Death population, black South Africans accounted for 79% of the population but had an HIV prevalence of 51%. The concerning factor is that there are such large numbers of HIV-positive cases being admitted to the Pretoria MLL and that HIV is not being routinely tested for in the mortuary setting.

In contrast to the study by Christiansen in which he found the top three causes of SUU-Death – cardiovascular, malignancy and infection, the majority of our cases died from respiratory disease processes followed by cardiovascular disease. TB accounted for 12 of the 31 respiratory disease-related deaths. In South Africa’s Millennium Development Goals, Goal 6 declares that there is a co-infection rate of 70% with TB and HIV.^21^ In a report by Statistics South Africa in 2009, TB accounted for 12% of all deaths, influenza and/or pneumonia 7.5% and HIV 3.1%,^22^ thus giving us a context for this difference in profile.

TB–HIV co-infection and antiretroviral treatment side effects are two possible explanations that warrant further investigation in this subpopulation. Tseng et al. indicated that sudden cardiac deaths in HIV-positive individuals accounted for 86% of all cardiac deaths with a mean rate of 2.6 per 1 000 person-years.^6^ In our study, cardiovascular diseases accounted for 17% of the cases with the majority being due to cardiomyopathy.

SUU-Deaths are a challenging autopsy and need to be approached with caution and the right amount of scepticism. The purpose of these autopsies is firstly to rule out an unnatural cause of death and secondly to provide, if possible, the cause of death even if it is natural.

The value of post-mortem HIV testing as an ancillary investigative technique to assist in the decision-making process and diagnosis of the cause of death should be further investigated. These cases are often admitted with no available history as the next of kin is not known or the medical history is incomplete. An HIV test can be helpful in determining the cause of death.

Unfortunately in South Africa, HIV has not been routinely tested for in the SUU-Death cases, and as a result statistics and observations regarding this challenging autopsy population have left a number of unanswered questions. This research study has attempted to address these questions and has shown that the analysis and observations provided by this testing provide an accurate well-documented source of information to facilitate the complex decisions that need to be taken in determining the cause of death and ultimately in our fight with regard to the HIV pandemic.

If medical records, post-mortem HIV test results and eyewitness testimony are made available before autopsy, a death scene investigator (DSI) can collect the relevant records and information and present them to the forensic pathologist along with the HIV test results. This would simplify the post-mortem examination for the forensic pathologist and facilitate a possible reduction in the number of SUU-Death cases admitted to the Pretoria MLL for a full autopsy. A reduction in SUU-Death cases could save the FPS a considerable amount of money and decrease the workload. This will ultimately streamline the provision of FPSs in the Pretoria MLL. A spin-off of testing for HIV routinely would be that there is a database of results that could be used to better understand HIV in the PM setting.

## Conclusion

SUU-Deaths accounted for 14% of all cases admitted to the Pretoria MLL. The HIV prevalence in the SUU deaths was 43%, which is 17% higher than the general mortuary population in Pretoria (*p* = 0.0045*)*. The majority of these deaths were due to respiratory disease processes with 12 cases having HIV–TB co-infection.

This study demonstrates the value of testing the SUU-Death population for HIV at autopsy. Further research recommendations are that research should be conducted in the implementation of routine HIV testing and testing for which stage of infection the HIV-positive SUU-Death cases are in. Screening for resistance and antiretrovirals could also be undertaken to determine if the person was on treatment at the time of death.
